# Protective Effect of Ferulic Acid on Lipopolysaccharide-Induced BV2 Microglia Inflammation via AMPK/mTOR Signaling Pathway

**DOI:** 10.3390/molecules28083482

**Published:** 2023-04-14

**Authors:** Xingru Chen, Xiaolan Zhou, Xiaoqing Cheng, Liting Lin, Qi Wang, Ruoting Zhan, Qingguang Wu, Sijun Liu

**Affiliations:** 1 School of Pharmaceutical Sciences, Guangzhou University of Chinese Medicine, Guangzhou 510006, China; cxr18997574312@163.com (X.C.); zhouxiaolan2002@163.com (X.Z.); m13727718977@163.com (X.C.); xshan7440@gmail.com (L.L.); zhanrt@gzucm.edu.cn (R.Z.); 2 Science and Technology Innovation Center, Guangzhou University of Chinese Medicine, Guangzhou 510006, China; wangqi@gzucm.edu.cn; 3 Key Laboratory of Chinese Medicinal Resource from Lingnan, Guangzhou University of Chinese Medicine, Ministry of Education, Guangzhou 510006, China

**Keywords:** BV2 microglia, ferulic acid, autophagy, AMPK/mTOR signaling pathway, NLRP3 inflammasome, neuroinflammation

## Abstract

In neurodegenerative diseases, microglial activation and neuroinflammation are essential for the control and progression of neurodegenerative diseases. Mitigating microglium-induced inflammation is one strategy for hindering the progression of neurodegenerative diseases. Ferulic acid (FA) is an effective anti-inflammatory agent, but its potential role and regulation mechanism in neuroinflammatory reactions have not been fully studied. In this study, the neuroinflammation model was established by lipopolysaccharide (LPS), and the inhibitory effect of FA on neuroinflammation of BV2 microglia was studied. The results showed that FA significantly reduced the production and expression of reactive oxygen species (ROS), tumor necrosis factor-α (TNF-α), leukocyte-6 (IL-6) and interleukin-1β (IL-1β). We further studied the mechanism of FA’s regulation of LPS-induced BV2 neuroinflammation and found that FA can significantly reduce the expression of mTOR in BV2 microglia induced by LPS, and significantly increase the expression of AMPK, indicating that FA may have an anti-inflammatory effect by activating the AMPK/mTOR signaling pathway to regulate the release of inflammatory mediators (such as NLRP3, caspase-1 p20 and IL-1β). We further added an autophagy inhibitor (3-MA) and an AMPK inhibitor (compound C, CC) for reverse verification. The results showed that FA’s inhibitory effects on TNF-α, IL-6 and IL-1β and its regulatory effect on AMPK/mTOR were destroyed by 3-MA and CC, which further indicated that FA’s inhibitory effect on neuroinflammation is related to its activation of the AMPK/mTOR autophagy signaling pathway. In a word, our experimental results show that FA can inhibit LPS-induced neuroinflammation of BV2 microglia by activating the AMPK/mTOR signaling pathway, and FA may be a potential drug for treating neuroinflammatory diseases.

## 1. Introduction

Microglia are immune cells in the central nervous system (CNS) that account for 10% of all cells in the CNS. During the inflammatory response, microglia are among the first immune cells to acquire immune activity. After inflammation causes brain damage, microglia become activated and initiate the inflammatory cascade in response to external or internal stimuli, producing inflammatory factors, and an overactive inflammatory response will result in brain injury [1]. TNF-α, IL-1β, IL-6 and ROS are neurotoxic mediators and inflammatory cytokines secreted by activated microglia that accelerate neurodegeneration and neural death and cause abnormal neural alterations [2,3].

Autophagy is an important mechanism for keeping neurons and glial cells healthy. Autophagy is a process that is controlled by several autophagy-related genes (ATGs) and signaling pathways. The AMP-activated protein kinase (AMPK)/mammalian target of rapamycin (mTOR) signaling pathway is at the heart of regulation. AMPK is a sub-cellular power metabolism sensor that regulates the energy balance through a range of techniques, including autophagy and protein anabolism. Its activation inhibits mTOR while increasing autophagy. LPS is a bacterial endotoxin that can induce the release of inflammatory mediators, activate microglia, and cause inflammatory reactions. Additionally, it can inhibit auto-phagosome formation and induce microglia to produce neuroinflammation by activating the mTOR signaling pathway [4]. Autophagy has also been shown to inhibit the expression of inflammatory factors induced by LPS [5]. Autophagy can regulate inflammatory cytokine secretion by inhibiting the inflammation pathway. Inflammation involves cytoplasmic complexes that recognize microorganisms and regulate the release of inflammatory cytokines. As an abnormally activated small inflammations cause excessive inflammatory-factor release, inflammation must be strictly controlled. Nod-like receptor protein 3 (NLRP3), produced in the inflammatory corpuscle, is a member of the NOD-like receptor family, which mediates the activation of pro-caspase-1. The activated pro-caspase-1 cleaves the precursor of IL-1β and converts it into mature IL-1β, which leads to the release of inflammatory factors [6]. Previous research has found that both ROS activation and autophagy perform a role in microglial activation. Down-regulation of the autophagy proteins microtubule-associated protein1 light chain3 (LC3) and Beclin1 causes mitochondrial dysfunction and ROS accumulation, which causes NLRP3 to release IL-1β and increases inflammation and neurotoxicity, resulting in neurodegeneration and neuronal death. By promoting mitosis, quercetin reduces ROS accumulation and relieves NLRP3 inflammation activation and IL-1β production [7]. As a result, inhibiting NLRP3 inflammation by modulating autophagy has been considered a targeted therapy for neurodegenerative diseases.

FA has numerous therapeutic properties, ranging from antioxidant to anti-inflammatory properties. Previous research has shown that FA can improve the learning and memory abilities of APP/PS1 transgenic mice, reduce neuroinflammation and decrease levels of TNF-α and IL-1β [8]. Long-term administration of FA can protect mice from Aβ-induced learning and memory deficits in vivo [9], and neurons from Aβ-induced oxidative stress and neurotoxicity [10]. However, the mechanism of how FA affects LPS-induced microglial inflammation has only been investigated in a few studies. In this in vitro study, we started to explore how FA affects LPS-induced inflammation in BV2 microglia.

## 2. Results

### 2.1. Effects of LPS and FA on the Viability of BV2 Microglia

**Figure 1:**
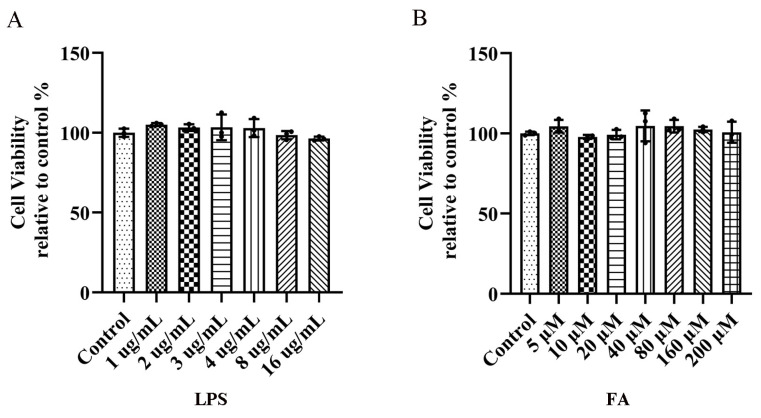
The effects of LPS and FA on BV2 microglial viability. (**A**) Effects of different concentrations of LPS on cell viability. (**B**) Effects of different concentrations of FA on cell viability. Data are means ± standard deviations (SDs) (*n* = 3).

We studied the effect of LPS on the activity of BV2 microglia. The results of the 3-(4, 5-dimethylthiazol-2-yl)-2,5-diphenyltetrazole (MTT) assay showed that LPS (1–16 μg/mL) had no significant effect on cell viability after 12 h compared with the control group (Figure 1A).

To verify the effect of FA on BV2 microglia, the activity of BV2 microglia treated with FA (0–200 μM) for 12 h was examined. The results of the MTT assay showed that the addition of FA (0–200 μM) for 12 h had no obvious effect on cell viability (Figure 1B). In the follow-up experiment, FA (40, 80, and 160 μM) was selected for experiments.

### 2.2. FA Inhibited LPS-Induced Oxidative Stress

**Figure 2:**
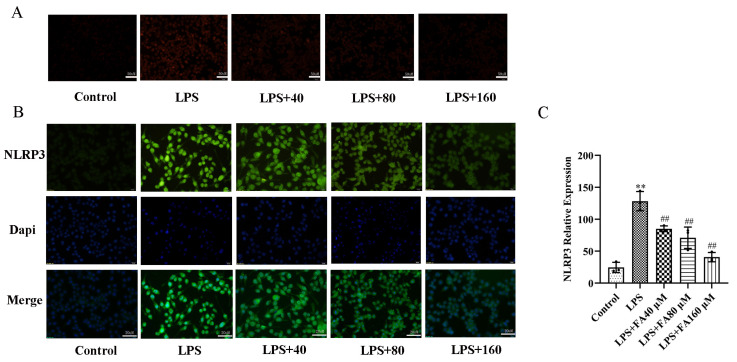
FA inhibited the LPS-induced ROS and NLRP3 (green) expression in a dose-dependent manner. (**A**) The expression of ROS (20× magnification) was observed under a fluorescence microscope. DHE staining results. Scale bar = 50 μM. (**B**) The expression of NLRP3 (50× magnification) in BV2 microglia was observed via inverted fluorescence microscope. (**C**) This figure shows the results of fluorescence statistics of NLRP3. The nuclei were stained with Hoechst 33,342 (blue). Scale bar = 20 μM. Data are means ± standard deviations (SDs) (*n* = 3). ** *p* < 0.01, vs. control; ^##^ *p* < 0.01, vs. LPS.

To observe whether FA can reduce ROS levels in BV2 microglia, we used a dihydroethidium (DHE) kit to detect the effect of FA on intracellular ROS levels in LPS-induced cells. The results showed that, compared with the control group, the red fluorescence intensity in the cells of LPS group was significantly enhanced, indicating that the ROS content in the cells of LPS group was increased. The red fluorescence intensity of the FA + LPS group was significantly weaker than that of the LPS group, suggesting that FA could inhibit the production of ROS in BV2 microglia induced by LPS. FA (160 μM) reduced the ROS expression level to nearly the same level as that of the control group (Figure 2A). These results indicate that FA inhibited LPS-induced ROS production in BV2 microglia by dose and concentration.

ROS is key to activating the NLRP3 inflammasome, and we have shown in the previous step that LPS can cause ROS levels to rise. Then immunocytochemistry (ICC) was used to investigate whether FA could reduce the expression of LPS-induced NLRP3 inflammation. ICC staining showed that NLRP3 fluorescent green expression in BV2 microglia was significantly increased after LPS treatment for 12 h (Figure 2B,C). FA + LPS significantly down-regulated the green fluorescence level of NLRP3 in a dose-dependent manner. In particular, FA (160 μM) reduced NLRP3 expression to close to the control level (Figure 2B,C).

### 2.3. Effect of FA on Pro-Inflammatory Cytokines in LPS-Induced BV2 Microglia

**Figure 3:**
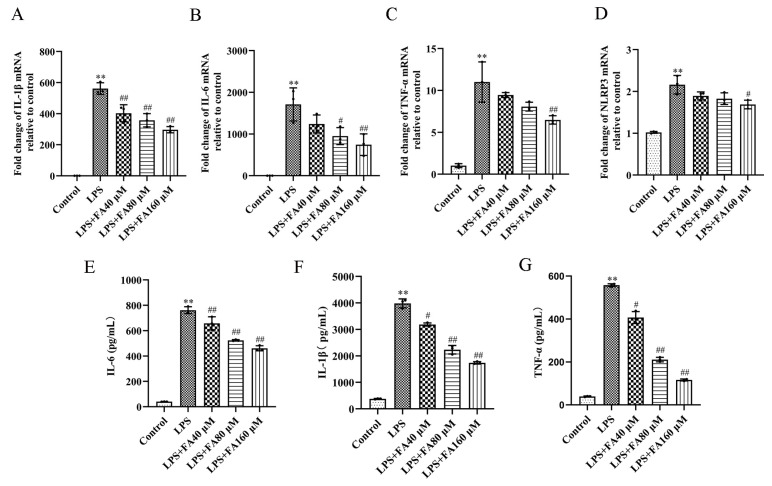
Effects of FA on the production of LPS-activated inflammatory cytokines in BV2 microglia BV2 microglia pretreated with different doses of FA for 2 h and then treated with 1 μg/mL of LPS for 12 h. (**A**–**D**) The expression levels of IL-1β, IL-6, TNF-αand NLRP3 were detected by reverse transcription-real-time quantitative PCR (qPCR). (**E**–**G**) The expression levels of IL-6, IL-1β and TNF-α in the supernatants left by BV2 microglia were determined by ELISA. Data are means ± standard deviations (SDs) (*n* = 3). ** *p* < 0.01, vs. control; ^##^ *p* < 0.01, ^#^ *p* < 0.05, vs. LPS.

The results of quantitative real-time PCR (qPCR) showed that compared with the control group, the expression levels of IL-1β, IL-6, TNF-α and NLRP3 in LPS group were significantly increased. The up-regulation rate of IL-6 was the highest (1710.420 ± 396.909). NLRP3 expression was the least up-regulated (2.161 ± 0.220). Compared with the LPS group, mRNA levels of inflammatory cytokines were significantly down-regulated in the FA + LPS group in a dose-dependent manner (Figure 3A–D). The results show that FA could improve the expression of inflammation induced by LPS dose-dependently, which would be beneficial to the treatment of inflammation.

The results of the enzyme-linked immunosorbent assay (ELISA) showed that compared with the control group, the LPS group significantly increased the expression of IL-6, IL-1β and TNF-α after 12 h of stimulation of BV2 microglia (Figure 3E–G). FA + LPS significantly decreased inflammation in a dose-dependent manner (Figure 3E–G), without causing any cytotoxicity to BV2 microglia. These results indicate that FA could inhibit the production of inflammatory factors induced by LPS.

### 2.4. FA Can Reduce the Levels of Inflammatory Proteins in LPS-Treated BV2 Microglia, Restore Autophagy and Activate the AMPK/mTOR Pathway

**Figure 4:**
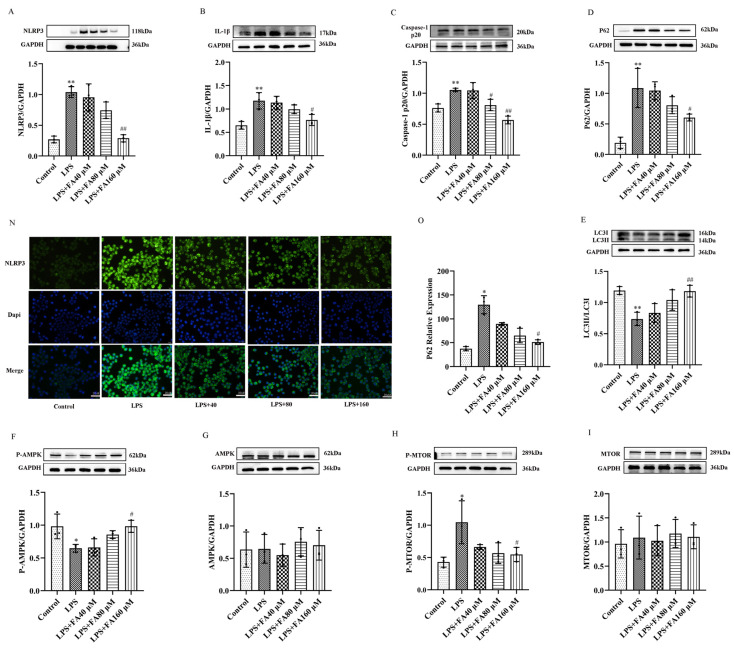
FA inhibits pro-inflammatory mediators and associated key targets in LPs-induced BV2 microglia. (**A**–**C**) NLRP3, IL-1β and caspase-1 p20 were detected by Western blot. FA restored the autophagy of BV2 microglia inhibited by LPS. (**D**,**E**) Autophagy-related proteins P62 and LC3 were detected by Western blot. (**F**–**I**) Western blot analysis showed the expression of P-AMPK/GAPDH, AMPK/GAPDH, P-mTOR/GAPDH and mTOR/GAPDH proteins. (**N**,**O**) The expression of NLRP3 (50× magnification) in BV2 microglia was observed via inverted fluorescence microscope. The nuclei were stained with Hoechst 33,342 (blue). Scale bar = 20 μM. Data are means ± standard deviations (SDs) (*n* = 3). ** *p* < 0.01, * *p* < 0.05, vs. control; ^##^ *p* < 0.01, ^#^ *p* < 0.05, vs. LPS.

In this study, Western blot (WB) was used to detect the expression levels of target proteins. The results showed that compared with the control group, the expression levels of NLRP3, IL-1β, caspase-1 p20 and other inflammatory proteins in LPS group were higher than those in the control group (Figure 4A–C); the expression of autophagy protein P62 was increased; and the expression of LC3 was decreased (Figure 4D,E). The P-AMPK protein decreased (Figure 4F), and AMPK did not show any change (Figure 4G). P-MTOR expression was increased (Figure 4H), whereas mTOR’s did not change (Figure 4I). Compared with the LPS group, the expression levels of NLRP3, IL-1β, caspase-1 p20 and other inflammatory proteins in medium- and high-dose FA groups decreased as the dose increased (Figure 4A–C). The expression of autophagy protein P62 decreased as the dose increased, and the expression of LC3 increased as the dose increased (Figure 4D,E). The protein P-AMPK’s expression increased with the dose (Figure 4F), and AMPK did not show any change in expression (Figure 4G). P-mTOR expression decreased with increases in dose (Figure 4H), and mTOR expression did not change (Figure 4I).

In this study, ICC was used to detect the expression of autophagy protein P62. The results showed that the LPS group could significantly improve the green fluorescence level of P62 in BV2 microglia; that is, LPS could induce the accumulation of P62 in BV2 microglia (Figure 4N,O). FA + LPS could reduce the green fluorescence level of P62 in BV2 microglia induced by LPS (Figure 4N,O), indicating that FA could increase autophagy flux at the gene and protein levels and inhibit the expression of inflammatory proteins through the AMPK/mTOR pathway.

### 2.5. Autophagy Inhibitors (3-MA) Block the Neuroprotective Effect of FA

**Figure 5:**
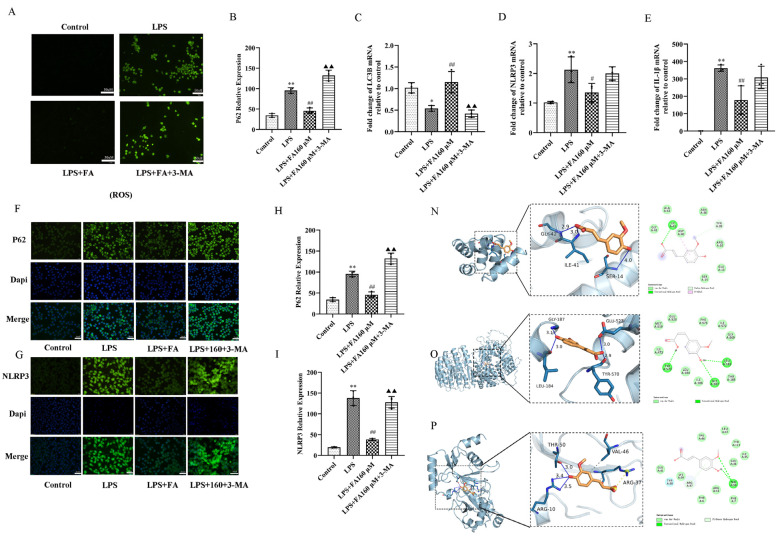
Autophagy inhibitors (3-MA) block the protective effects of FA: restoring autophagy and inhibiting inflammation levels. (**A**) ROS was detected via DCF-DA fluorescent probe. (**B**–**E**) The qPCR kit was used to detect autophagy-related proteins P62 and LC3; and NLRP3 and IL-1β. (**F**–**I**) The expression of NLRP3 and P62 (50× magnification) in BV2 microglia was observed via inverted fluorescence microscope. The nuclei were stained with Hoechst 33,342 (blue). Scale bar = 20 μM. (**N**–**P**) Molecular docking results of ferulic acid with LC3, NLRP3 and P62 proteins. Data are means ± standard deviations (SDs) (*n* = 3). ** *p* < 0.01, * *p* < 0.05, vs. control; ^##^ *p* < 0.01, ^#^ *p* < 0.05, vs. LPS; ^▲▲^ *p* < 0.01, vs. FA + LPS.

To verify the interaction between FA’s inhibition of inflammation and autophagy, autophagy inhibitor (3-MA) was added to FA + LPS. The results showed that ROS expression was significantly increased in the LPS + FA + 3-MA group, indicating that 3-MA could destroy the inhibitory effect of FA on ROS (Figure 5A). qPCR results showed that NLRP3 and IL-1β mRNA expression levels were increased, LC3 was inhibited, P62 expression was increased and autophagy was blocked in the LPS + FA + 3-MA group (Figure 5B–E). ICC results showed that the green fluorescence levels of NLRP3 and P62 increased after the addition of 3-MA (Figure 5F–I). In addition, molecular binding results showed that FA had good binding forces with the target proteins, such as NLRP3, LC3 and P62, further indicating that autophagy plays a key role in inhibiting inflammation (Figure 5N–P).

### 2.6. Autophagy Inhibitors (3-MA) Restore the Effects of LPS on Inflammatory Cytokines, Autophagy and the AMPK/mTOR Pathway

**Figure 6:**
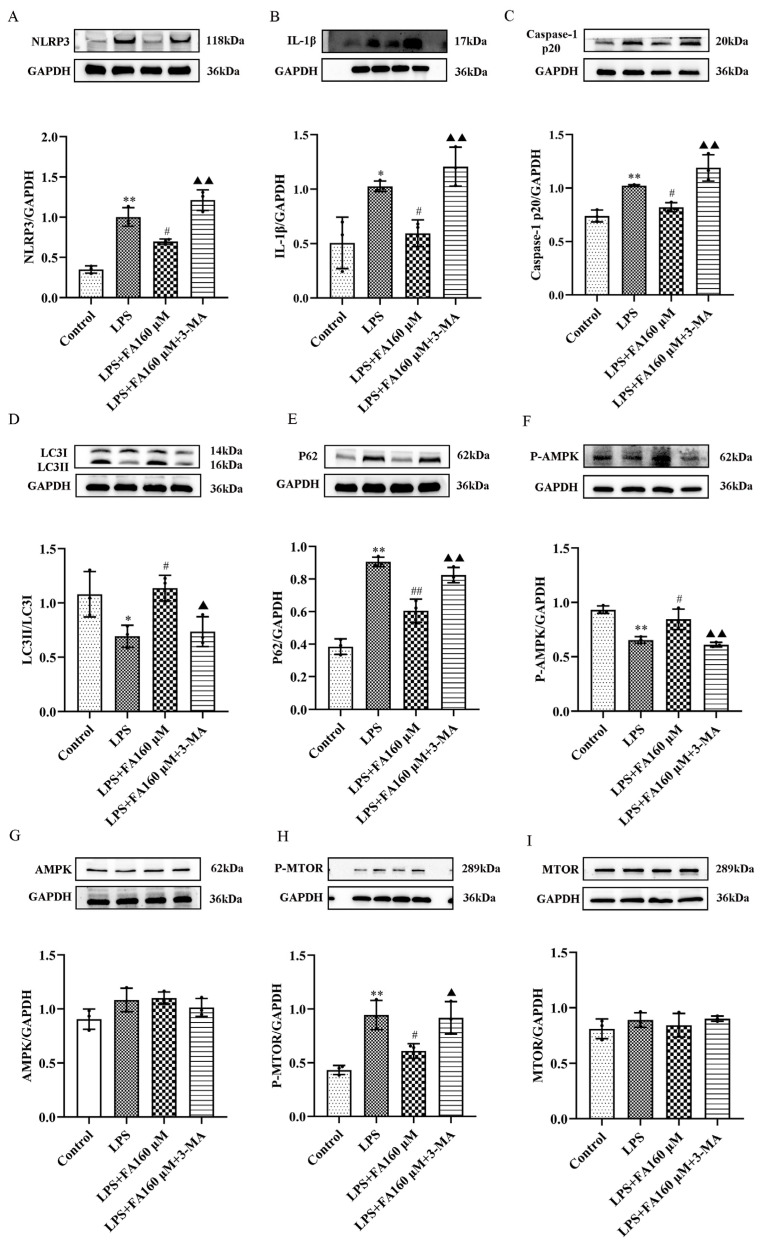
Autophagy inhibitors (3-MA) undermine all beneficial effects of FA. (**A**–**I**) NLRP3, IL-1β and caspase-1 p20 were detected by Western blot. Autophagy-related proteins LC3 and P62 and pathway proteins P-AMPK/GAPDH, AMPK/GAPDH, P-mTOR/GAPDH and mTOR/GAPDH were expressed. Data are means ± standard deviations (SDs) (*n* = 3). ** *p* < 0.01, * *p* < 0.05, vs. control; ^##^ *p* < 0.01, ^#^ *p* < 0.05, vs. LPS; ^▲▲^ *p* < 0.01, ^▲^ *p* < 0.05 vs. FA + LPS.

In this study, WB was used to detect the expression levels of key proteins. The results showed that compared with the control group, the expression levels of NLRP3, IL-1β, caspase-1 and p20 in the LPS group and 3-MA group were significantly increased (Figure 6A–C); that of LC3 was decreased; and that of the autophagy protein P62 was increased (Figure 6D,E). P-AMPK was down-regulated (Figure 6F), and AMPK did not change (Figure 6G). P-mTOR expression increased (Figure 6H), and mTOR expression did not change (Figure 6I). Compared with the LPS group, the expression levels of NLRP3, IL-1β and caspase-1 p20 in the FA + LPS group decreased as the dose increased (Figure 6A–C). The expression of autophagy protein P62 decreased as the dose increased, and the expression of LC3 increased with the dose (Figure 6D,E). The protein P-AMPK increased with the dose (Figure 6F), and AMPK did not change (Figure 6G). P-mTOR decreased with the dose (Figure 6H), and the mTOR protein did not change (Figure 6I). FA had no recovery effect on 3-MA. These results suggest that adding autophagy inhibitors can disrupt the beneficial effects of FA, indicating that autophagy is very important for FA, and FA may reduce the production of inflammatory proteins through autophagy.

### 2.7. AMPK Inhibitors Block the Protective Effect of Neuroinflammation Induced by FA

**Figure 7:**
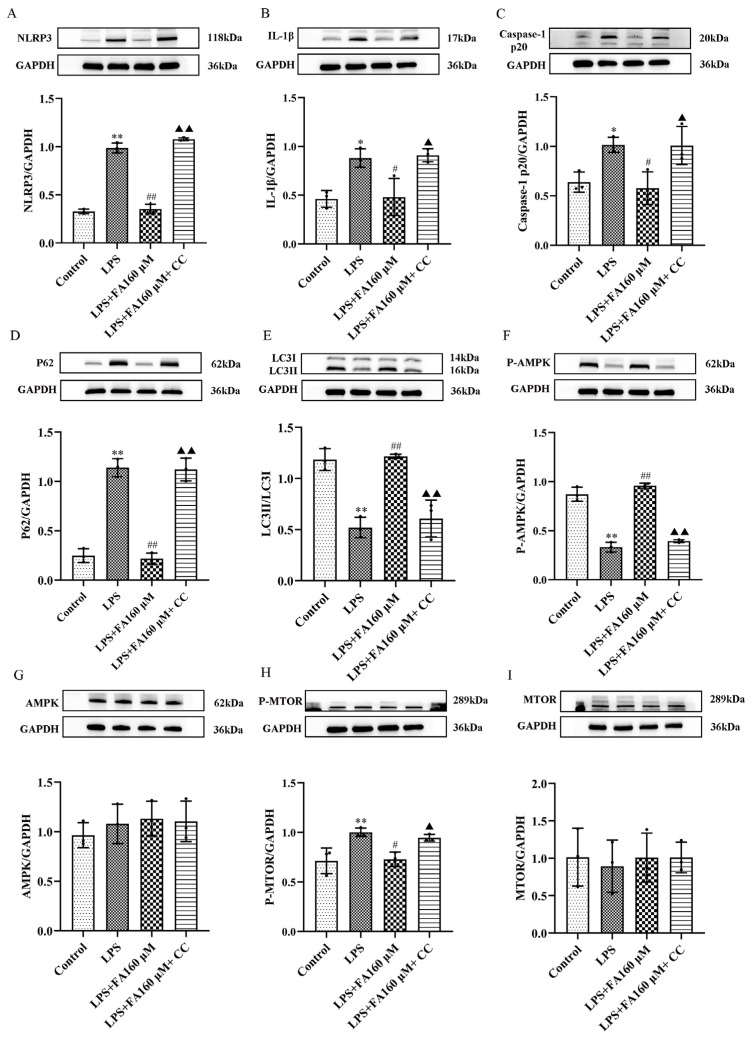
AMPK inhibitors destroy all beneficial effects of FA. (**A**–**I**) NLRP3, IL-1β and caspase-1 p20 were detected via Western blot. Autophagy-related proteins LC3 and P62 and pathway proteins P-AMPK/GAPDH, AMPK/GAPDH, P-MTOR/GAPDH and mTOR/GAPDH were expressed. Data are means ± standard deviations (SDs) (*n* = 3). ** *p* < 0.01, * *p* < 0.05, vs. control; ^##^ *p* < 0.01, ^#^ *p* < 0.05, vs. LPS; ^▲▲^ *p* < 0.01, ^▲^ *p* < 0.05 vs. FA + LPS.

In this study, we used the AMPK inhibitor CC to reverse evidence that FA inhibits inflammation through the AMPK/mTOR signaling pathway. The results showed that the expression levels of NLRP3, IL-1β, caspase-1 p20 and other inflammatory proteins in the LPS + FA + CC group were higher than those in the control group (Figure 7C). The autophagy protein P62 was increased, LC3 was decreased (Figure 7D,E), the protein P-AMPK was decreased (Figure 7F) and AMPK was unchanged (Figure 7G). P-mTOR increased (Figure 7H), and mTOR did not change (Figure 7I). Compared with the LPS group, the expression levels of NLRP3, IL-1β, caspase-1 p20 and other inflammatory proteins in the FA + LPS group decreased as the dose increased (Figure 7A–C). The expression of the autophagy protein P62 was decreased as the dose increased, and the expression level of LC3 increased as the dose increased (Figure 7D,E). The expression of the protein P-AMPK increased as the dose increased (Figure 7F), and the AMPK protein’s expression did not change (Figure 7G). P-mTOR expression decreased as the dose increased (Figure 7H), and mTOR expression did not change (Figure 7I). FA + LPS had no recovery effect on the CC group. These results suggest that the addition of the AMPK inhibitor CC can disrupt the beneficial effects of FA, suggesting that FA may act through the AMPK/mTOR signaling pathway. Inhibition of the AMPK/mTOR signaling pathway can lead to elevated expression levels of inflammatory proteins, and the AMPK/mTOR signaling pathway plays a key role in reducing inflammation.

In summary, our in vitro results showed that FA can reduce the expression of inflammatory proteins, and FA can also restore the autophagy of BV2 microglia, which may be related to the activation of the AMPK/mTOR pathway.

## 3. Discussion

FA is widely used in free radical scavenging, in antioxidation and by anti-inflammatory factors. In vitro, FA has been shown to have a significant inhibitory effect on the formation of inflammatory factors. FA treatment of benzo(a) pyrene-induced microglia, for example, can reduce neuroinflammation and the release of cellular inflammatory factors such as IL-6, IL-1β, NO and ROS [11], indicating that FA can play a neuroprotective role by inhibiting the microglium-mediated pro-inflammatory response. In this study, we investigated the mechanism of FA’s anti-neuroinflammatory effect in vitro. The results showed that FA can reduce the neuroinflammatory response induced by lipopolysaccharide, which may be related to the restoration of autophagy and activation of the AMPK/mTOR pathway.

LPS is one of the Gram-negative bacterium’s immunostimulatory components [12]. Microglia are the central nervous system’s first immune barrier and play an important role in inflammation. LPS can recognize and bind microglial Toll-like receptors, activating them, changing their morphology and promoting the release of microglial neuroinflammatory factors (TNF-α, IL-1β and IL-6) [13]. In this study, LPS-induced BV2 microglia produced TNF-α, IL-1β and IL-6, and increased NLRP3 expression, suggesting that microglia activation was involved in LPS-induced neuroinflammation. This observation is similar to that reported by Yongjuan Liu et al. [14]. These results suggest that FA can inhibit the inflammatory response of BV2 induced by LPS and may act as a neuroprotective agent.

Inflammation is an immune response that occurs as a result of tissue damage or invading pathogens. When central nervous system injury and inflammatory cytokines are present, the main event of the neuroinflammatory response is activation of the NLRP3 inflammatory body. Excessive activation of NLRP3 inflammatory bodies impairs microglial autophagy and worsens neurodegenerative diseases [15,16,17]. Several studies have shown a relationship between NLRP3 inflammatory bodies and neurodegenerative diseases, including Parkinson’s disease (PD) and AD [18,19]. Studies have shown that ROS can increase the activation of NLRP3 and caspase-1 and the release of IL-1β in macrophages [20]. Subsequent experiments have shown that adding ROS inhibitors can significantly reduce IL-1β production in microglia [21], suggesting that the production of inflammatory factors may be related to oxidative stress. In this study, LPS was found to increase the ROS content in BV2 microglia, and subsequently, the expression levels of NLRP3 mRNA and protein were also increased by qPCR and immunofluorescence, indicating that LPS could activate the NLRP3 inflammatory body, and FA reduced NLRP3 expression.

The inflammasome is a multi-protein complex made up of NLRP3, ASC and pro- caspase-1. It induces cell death under pathological inflammatory and cellular-stress conditions [22,23]. The pyrrole domain (PYD) of NLRP3 binds to ASC after activation, and pre-caspase-1 is recruited via the caspase recruitment domain (CARD) [24]. The original caspase-1 is then split into caspase-1 p10 and caspase-1 p20 by NLRP3 inflammatory bodies, and mature caspase-1 releases IL-1β. Overexpression of this inflammatory cytokine amplifies the central nervous system’s inflammatory response and significantly promotes neuronal damage [25]. FA decreases the expression of NLRP3, caspase-1 and IL-1β in BV2 microglia and inhibits caspase-1 activation and IL-1β maturation, according to the findings of this study. These findings suggest that FA may suppress TNF-α, IL-6 and IL-1β expression by inhibiting the activation of NLRP3 inflammatory bodies.

Autophagy has also been linked to inflammation, which is mediated by NLRP3 inflammatory bodies. Autophagy, the key mechanism of NLRP3 activation, inhibits the production of inflammatory factors via redox balancing of ROS [26]. The destruction of microglial autophagy in the AD model can exacerbate neuronal damage [27]. Autophagy may regulate LPS-stimulated microglial inflammation and neurotoxicity. This study looked into the effect of FA on autophagy in BV2 microglia. The results showed that FA activated autophagy by increasing LC3 expression and decreasing P62 expression. To better understand the relationship between autophagy and inflammation, 3-MA autophagy inhibitors were chosen for further testing. The results showed that the autophagy inhibitor 3-MA increased TNF-α, IL-6 and IL-1β. The decreases in NLRP3 expression and IL-1β level induced by FA could be partially blocked by autophagy inhibitors.

The AMPK/mTOR signaling pathway has become an important autophagy regulator. Studies have shown that LPS inhibits autophagy formation and induces neuroinflammation in microglia by activating the mTOR pathway [28,29]. In this study, the AMPK inhibitor was used for validation. We found that FA can increase AMPK phosphorylation and inhibit mTOR phosphorylation, suggesting that FA can increase autophagy through this pathway. FA obstruction of NLRP3 and IL-1β overexpression can be partially blocked by AMPK inhibitors, suggesting that AMPK/mTOR pathway is closely related to NLRP3 inflammatory bodies. These results suggest that FA can enhance autophagy and reduce inflammatory protein expression through the AMPK/mTOR signaling pathway.

## 4. Materials and Methods

### 4.1. Cell Culture

BV2 microglia (CL-0493) were purchased from Procell Life Science & Technology Co., Ltd. (Wuhan, China) BV2 was cultured in DMEM containing 1% penicillin and 10% fetal bovine serum (Gibco, Gaithersburg, PA, USA), in a humidified incubator with 5% CO_2_ at 37 °C.

### 4.2. Cell Treatment

FA (product number: A0050; purity: 99.96%) was purchased from Chengdu Must Bio-Technology Co., Ltd. LPS (L2880, Sigma, St. Louis, MO, USA). The concentrations of FA and LPS were determined by MTT assay. BV2 microglia were treated with FA (40, 80, 160 μM) for 2 h and LPS (1 μg/mL) for 12 h, respectively. 3-MA (D426448, 5 mM, Aladdin, Shanghai, China) was added to the cells 1 h before FA was added, and the subsequent experimental procedures were the same as before. CC (5142-23-4, 2 μM, MCE, West Sayville, NY, USA) was added to the cells 2 h before FA was added, and the subsequent experimental procedures were the same as before.

### 4.3. Cell Viability Assay

BV2 microglia (5 × 10^3^ cells/well) were inoculated into 96-well plates and treated with LPS (0–16 µg/mL) at different concentrations for 12 h. Cell viability was calculated using 3-(4,5-dimethylthiazol-2-yl)-2,5-diphenyltetrazole (MTT) assay. After 24 h of culturing in a constant-temperature cell incubator, the liquid was sucked clean with a pipette gun, FA/LPS was added, 5 multiple pores were set in each group and the plates were incubated in a constant-temperature cell incubator for 12 h. The medium containing the drug was removed, and the pre-prepared MTT working liquid (90% basic medium +10% MTT mother liquor) was added: 100 μL per well. After incubation at 37 °C for 4 h, the liquid was removed. DMSO was added to the well plate, 150 μL per well. The OD value was obtained by detecting the enzyme label at 490 nm. The cell viability (%) = (OD(Asample)-OD (medium))/(OD(ACON)-OD(medium)).

### 4.4. Levels of ROS

In this study, DCF-DA probe (S0033S, Beyotime, Beijing, China) and DHE fluorescence staining (S0063, Beyotime) were used to detect intracellular ROS levels. BV2 microglia at the logarithmic growth stage were inoculated into 12-well cell plates, incubated for 12 h and given LPS and FA. After 12 h, the plates were removed from the cell incubator and fixed with 4% paraformaldehyde for 15 min. The residual liquid was cleaned with PBS, then basic medium containing DCFH-DA (10 μM) was added and the plates were incubated at 37 °C for 30 min in the dark. After incubation, the residual extracellular DCFH-DA solution was cleaned with PBS. The fluorescence intensity was observed by inverted fluorescence microscope (Nikon, Tokyo, Japan, TE2000-U). The average fluorescence intensity of three random fields was quantified by ImageJ 1.51 software. DHE is a cell penetrating dye that is oxidized by superoxide to fluorescent ethidium bromide and embedded in DNA. The final concentration of 10 μM was diluted with DMEM medium at a ratio of 1:1000 in a super clean table. The treated BV2 microglial cells were added into the working solution of 100 μL (Nikon, TE2000-U) and cultured for 30 min at 37 °C in the dark. The cells were washed 3 times with pre-cooled PBS. Fluorescence intensity was measured via inverted fluorescence microscope. The average fluorescence intensity of three random fields was quantified by ImageJ 1.51 software.

### 4.5. Enzyme-Linked Immunosorbent Assay (ELISA)

To determine the levels of inflammatory cytokines (TNF-α, IL-6 and IL-β) in LPS and LPS+FA induced BV2 microglia, FA + LPS- and LPS-treated cell supernatants were collected. Then, levels of TNF-a (RX202412M, Ruixinbio, Quanzhou, China), IL-6 (RX203049M, Ruixinbio) and IL-1β (RX203063M, Ruixinbio) were determined with their corresponding ELISA kits, as instructed by the producers.

This ELISA kit uses a double-antibody-sandwich two-step method. Mouse TNF-α, IL-1β and IL-6 calibrators were added to the microcellular enzyme plate pre-coated with anti-mouse TNF-α antibody, anti-mouse IL-1β antibody and anti-mouse IL-6 antibody, and the samples to be tested were incubated at room temperature. Then biotin antibody was added, incubated and fully washed, and avidin was added. After over incubation and full washing, the unbound components were removed, and a sandwich complex of solid antibody-antigen-antibody was formed on the solid surface of the microporous plate. Substrate A and B were added to produce blue products. Under the action of the termination solution, the products were finally transformed into yellow. OD value was measured at the wavelength of 450 nm on the enzyme label instrument.

### 4.6. Quantitative Real-Time PCR (qRT-PCR)

BV2 microglia were inoculated in 6-well plates (1 × 10^5^ cells/well) and cultured for 12 h. FA was pretreated with different concentrations for 2 h and then treated with 1 μg/mL LPS for 12 h. Cells were collected according to the manufacturer’s instructions, and total RNA was extracted from the cells using EZ-press RNA Purification Kit reagents (B004D, EZBioscience, Roseville, MN, USA). mRNA was reverse transcribed into cDNA using a cDNA synthesis kit. The synthesized cDNA (1 μL) was mixed with specific primers. The relative expression levels of the target gene were evaluated by qPCR using denaturation at 95 °C for 15 s, annealing at 60 °C for 1 min, extension at 95 °C for 30 s and 40 cycles. GAPDH and β-actin were used as internal control gened (Table 1).

### 4.7. Molecular Docking

AutoDock Vina 1.1.2 was used to dock ligands and receptors to study the binding mode of FA with NLRP3 and P62. The NLRP3 and P62 protein structures were downloaded from the PDB database and visualized using Pymol. Mgtools 1.5.6 was used to calculate the charges of dehydration, hydrogenation and non-polar hydrogen bonding. Ligands and receptors were stored separately in pdbqt format. We downloaded the 3D structure in the active Ingredients FA sdf format from PubChem and imported it into ChemDraw 3D, where the MM2 module was used to minimize the energy, obtain the advantage concept of the lowest energy and save it in the mol2 file. The ligand was docked to the receptor using Autodock Vina 1.1.2 and visualized using PYMOL.

### 4.8. Western Blot

Drugs were added to the BV2 microglia as previously described. Twelve hours after administration, cells were collected and cleaved; protein concentration was quantified; protein lysate (25 μL) was electrophoresed and transferred onto PVDF membrane (pore size: 0.45 μm). After the transmembrane was completed, the PVDF membrane was sealed with 5% skim milk for 2 h and incubated with the specified primary antibody—against P-AMPK (T55608S, 1:1000, Abmart, Shanghai, China), mTOR(T55306S, 1:5000, Abmart), P-mTOR (T56571S, 1:5000, Abmart), LC3-II/LCI (T55992F, 1:500, Abmart), IL-1β (TA5103, 1:500, Abmart), P62 (T55546S, 1:5000, Abmart), NLRP3 (15101s, 1:1000, Cell Signaling Technology (CST), Danvers, MA, USA), GAPDH (T0004, 1:1000, Affinity, Cincinnati, OH, USA), caspase-1 p20 (AF4005, 1:1000, Affinity) or AMPK (AF6423, 1:1000, Affinity)—at 4 °C overnight. PVDF membrane and HRP-labeled secondary antibody were cultured at room temperature for 1 h and rinsed 3 times. Visualization of the protein bands was achieved with an enhanced chemiluminescence (ECL) kit (P0018FS, Vazyme, Nanjing, China) and imaging system (3500R, Tanon, Shanghai, China), whose grayscale values were calculated using ImageJ version 5.0.

### 4.9. Immunocytochemistry

BV2 microglia at the logarithmic growth stage were inoculated on 6-well cell cover slides and incubated for 24 h. Cells were added to FA for 2 h, removed from the incubator and then added to LPS. After 12 h, remove from the incubator for operation. After 12 h, the cells were removed from the incubator, fixed with 4% paraformaldehyde and infiltrated with 0.3% Triton. P62 (T55546S, 1:50, Abmart, Shanghai, China) and NLRP3 (15101s, 1:100, Cell Signaling Technology (CST), Danvers, MA, USA) were incubated overnight at 4 °C—200 μL diluent per well. The nuclei were stained with DAPI (C0065, Solarbio, Beijing, China). Images were captured using a fluorescence microscope (OLYMPUS BX53). ImageJ was used to locate and visualize P62 and NLRP3.

### 4.10. Statistical Analysis

GraphPad Prism 8.0 software was used for statistical analysis. The results of all in vitro tests were expressed as the mean ± SD of three independent experiments. After validation of normal distribution, data analysis was performed by one-way ANOVA and Turkey’s multiple test, and *p* < 0.05 was considered statistically significant.

## 5. Conclusions

FA inhibited pro-inflammatory cytokines released by BV2 microglia and reduced ROS production. In addition, FA restores autophagy and activates the AMPK/mTOR pathway in LPS-activated BV2 microglia, reducing NLRP3 inflammatory protein levels. These results suggest that FA could inhibit neuroinflammation, which may play a role in the treatment of neurodegenerative diseases.

## Figures and Tables

**Table 1:** Primer sequences of IL-6, IL-1β, TNF-α, NLRP3, P62, LC3B and β-actin.

Gene	Sense (5′-3′)	Anti-Sense (5′-3′)
IL-6	ACCTGTCTATACCACTTCACAAGT	AGAATTGCCATTGCACAACTCT
IL-1β	AGGAGAACCAAGCAACGACA	TGCTTGGGATCCACACTCTC
TNF-α	CTTCTCATTCCTGCTTGTGGCA	GAGGCCATTTGGGAACTTCTCAT
NLRP3	CAACAATGATCTTGGCGATCTGT	TACATTTCACCCAACTGTAGGCT
P62	CTTCGGAAGTCAGCAAACCTGA	TCCCGACTCCATCTGTTCCTC
LC3B	GATACAAGGGGGAGAAGCAGCT	CTGCAAGCGCCGTCTGATT
β-actin	GGTCATCACTATTGGCAACGAGC	CCAGACAGCACTGTGTTGGCATA

## Data Availability

The data presented in this study are available in the article.

## Sample Availability

Samples of the compounds are not available from the authors.
